# μ-2,3,5,6-Tetra­kis(pyridin-2-yl)pyrazine-bis­[(2,2′:6′,2′′-terpyridine)­ruthenium(II)] tetra­kis­(hexa­fluoridophosphate) acetonitrile tetra­solvate

**DOI:** 10.1107/S1600536812051215

**Published:** 2013-01-09

**Authors:** Hershel Jude, Brian L. Scott, Reginaldo C. Rocha

**Affiliations:** a Los Alamos National Laboratory, MPA Division, Los Alamos, NM 87545, USA

## Abstract

In the title compound [Ru_2_(C_15_H_11_N_3_)_2_(C_24_H_16_N_6_)](PF_6_)_4_·4CH_3_CN, two of the counter-ions and one of the solvent mol­ecules are disordered with occupancies for the major components between 0.57 (2) and 0.64 (1). The structure of the dinuclear tetracation exhibits significant distortion from planarity in the bridging 2,3,5,6-tetra­kis­(pyridin-2-yl)pyrazine (tppz) ligand, which has a saddle-like geometry with an average dihedral angle of 42.96 (18)° between adjacent pyridine rings. The metal–ligand coordination environment is nearly equivalent for the two Ru^II^ atoms, which have a distorted octa­hedral geometry due to the restricted bite angle [157.57 (13)–159.28 (12)°] of their two *mer*-arranged tridendate ligands [2,2′:6′,2′′-terpyridine (tpy) and tppz] orthogonal to each other. At the peripheral tpy ligands, the average Ru—N bond distance is 2.072 (4) Å for the outer N atoms *trans* to each other (N_outer_) and 1.984 (1) Å for the central N atoms (N_central_). At the bridging tppz ligand, the average metal–ligand distances are significantly shorter [2.058 (4) Å for Ru—N_outer_ and 1.965 (1) Å for Ru—N_central_] as a result of both the geometric constraints and the stronger π-acceptor ability of the pyrazine-centered bridge. The dihedral angle between the two tpy planes is 27.11 (6)°. The intra­molecular linear distance between the two Ru atoms is 6.6102 (7) Å.

## Related literature

For a previously reported solvent-free structure of this compound, see: Yoshikawa *et al.* (2011 ▶). For the crystal structure of a related diruthenium(II) compound containing the {(tpy)Ru(tppz)} moiety, see: Chen *et al.* (2011 ▶). For details of the synthesis, see: Arana & Abruña (1993 ▶); Rocha *et al.* (2008 ▶); Thummel & Chirayil (1988 ▶); Vogler *et al.* (1996 ▶); Wadman *et al.* (2009 ▶). For general properties of this compound, see: Arana & Abruña (1993 ▶); Dattelbaum *et al.* (2002 ▶); Flores-Torres *et al.* (2006 ▶); Gourdon & Launay (1998 ▶); Jones *et al.* (1998 ▶); Thummel & Chirayil (1988 ▶); Vogler *et al.* (1996 ▶); Wadman *et al.* (2009 ▶).
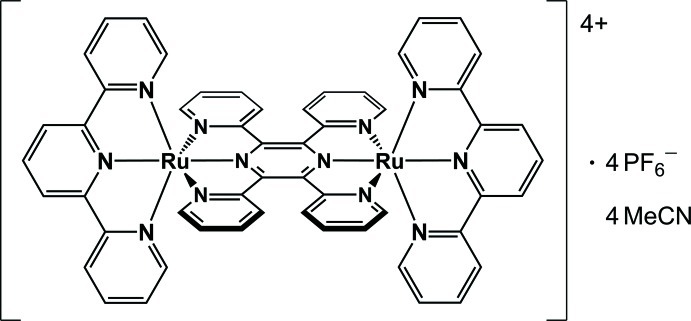


## Experimental

### 

#### Crystal data

[Ru_2_(C_15_H_11_N_3_)_2_(C_24_H_16_N_6_)](PF_6_)_4_·4C_2_H_3_N
*M* *_r_* = 1801.20
Monoclinic, 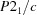

*a* = 11.8871 (9) Å
*b* = 31.824 (2) Å
*c* = 18.5168 (14) Å
β = 95.880 (1)°
*V* = 6968.0 (9) Å^3^
*Z* = 4
Mo *K*α radiation
μ = 0.64 mm^−1^
*T* = 120 K
0.18 × 0.10 × 0.08 mm

#### Data collection

Bruker D8 with APEXII CCD diffractometer
Absorption correction: multi-scan (*SADABS*; Sheldrick, 2008 ▶) *T*_min_ = 0.893, *T*_max_ = 0.950
67490 measured reflections
12753 independent reflections
8864 reflections with *I* > 2σ(*I*)
*R*_int_ = 0.107

#### Refinement

*R*[*F* ^2^ > 2σ(*F* ^2^)] = 0.045
*wR*(*F* ^2^) = 0.102
*S* = 1.10
12753 reflections
1101 parameters
78 restraints
H-atom parameters constrained
Δρ_max_ = 0.75 e Å^−3^
Δρ_min_ = −0.50 e Å^−3^

### 

Data collection: *APEX2* (Bruker, 2007 ▶); cell refinement: *SAINT-Plus* (Bruker, 2007 ▶); data reduction: *SAINT-Plus*; program(s) used to solve structure: *SHELXS97* (Sheldrick, 2008 ▶); program(s) used to refine structure: *SHELXL97* (Sheldrick, 2008 ▶); molecular graphics: *SHELXTL* (Sheldrick, 2008 ▶); software used to prepare material for publication: *publCIF* (Westrip, 2010 ▶).

## Supplementary Material

Crystal structure: contains datablock(s) I, global. DOI: 10.1107/S1600536812051215/zl2523sup1.cif

Structure factors: contains datablock(s) I. DOI: 10.1107/S1600536812051215/zl2523Isup2.hkl

Additional supplementary materials:  crystallographic information; 3D view; checkCIF report

## Figures and Tables

**Fig. 1.**
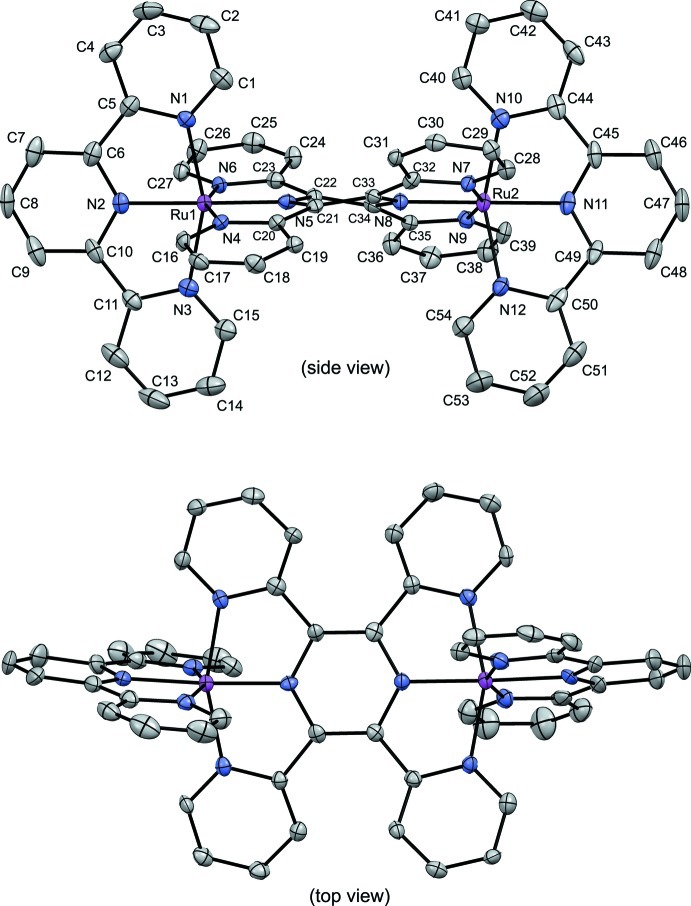
Two views of the single-crystal structure of the cation (I) in [(tpy)Ru(tppz)Ru(tpy)](PF6)4×4MeCN. Displacement ellipsoids are drawn at the 50% probability level. H atoms are omitted for clarity.

**Fig. 2.**
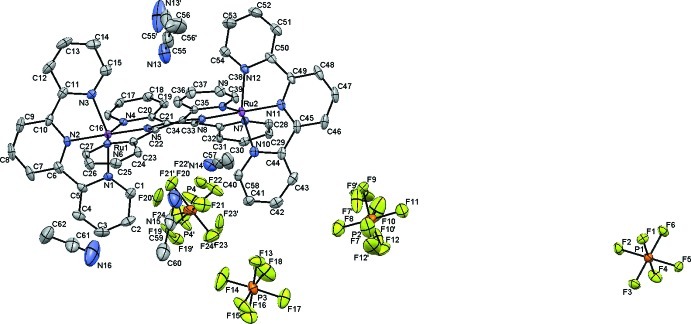
Single-crystal structure of [(tpy)Ru(tppz)Ru(tpy)](PF6)4×4MeCN, with all counterions and solvent molecules included. Also shown is the observed disorder in two of the PF6- anions (P2,F7–F12 and P4,F19–F24) and one of the MeCN solvent molecules (N13,C55,C56). Displacement ellipsoids are drawn at th e 50% probability level. H atoms are omitted for clarity.

## supplementary crystallographic information

### Comment

The PF_6_^-^ salt of the symmetric dinuclear complex [(tpy)Ru^II^(µ-tppz)Ru^II^(tpy)]^4+^ (I) in acetonitrile crystallized in the monoclinic space group (*P*2_1_/*c*). Its crystal structure is shown in Figs. 1 and 2, and discussed below.

A structure of the compound [(tpy)Ru(tppz)Ru(tpy)](PF_6_)_4_ was recently reported (Yoshikawa *et al.*, 2011). In this case, the compound crystallized in the triclinic (*P*1) space group, without containing solvent molecules in the unit. However, the relatively poor quality of that structure (*R*-factor = 15.61%) and relatively large deviations in metal-ligand bond distances (0.02 Å) and angles (0.6–0.8°) precludes an accurate comparison with the data reported here. A better comparative analysis involves the only other crystallographically characterized compound featuring the {(tpy)Ru(tppz)Ru} fragment, the PF_6_^-^ salt of the photocatalyst [(tpy)Ru^II^(tppz)Ru^II^(bpy)(Cl)]^3+^ (II; Chen *et al.*, 2011).

In the {(tpy)Ru(tppz)} moiety of II, the average Ru—N distances (tpy: Ru—N_outer_ = 2.071 (4) Å and Ru—N_central_ = 1.984 (4) Å; tppz: Ru—N_outer_ = 2.056 (4) Å and Ru—N_central_ = 1.963 (4) Å) and bite angles of the *mer*-coordinated tpy and tppz (157.59 (17)°–159.43 (16)°) are nearly identical to those observed for I. Also similar but even more pronounced in II is the highly distorted saddle-like conformation adopted by the bridging tppz ligand, with an average torsion angle of 52.2 (3)° between adjacent pyridyl rings.

In [(tpy)Ru(tppz)Ru(tpy)](PF_6_)_4_×4MeCN, the cation (I) packs in alternating layers with the PF_6_^-^ anions and solvent molecules packed between the cations. No significant interactions are present between different layers. Two of the PF_6_^-^ counterions and one of the MeCN solvent molecules are disordered (Fig. 2). The percentage of the major disordered component is 62 (2)% for the first anion (atoms P2, F7 to F12), 57 (2)% for the second anion (P4, F19 to F24), and 64 (1)% for the solvent molecule (N13, C55, C56).

### Experimental

The synthesis of [(tpy)Ru^II^(tppz)Ru^II^(tpy)](PF_6_)_4_ was performed by two methods. A) In this route, we took advantage of our previously reported precursor to tppz-bridged dimers, the mixed-valent solvento complex [(EtOH)Cl_2_Ru^II^(tppz)Ru^III^Cl_3_] (Chen *et al.*, 2011; Rocha *et al.*, 2008). This precursor was utilized in a reaction with 2 equiv of tpy in EtOH heated at reflux for 8 h, under an Ar atmosphere. Et_3_N in stoichiometric excess was added as a reductant. Following substitution of the Cl^-^ ligands, the tpy-capped dimer was collected as a solid salt by filtration of the precipitate formed upon addition of a concentrated aqueous solution of NH_4_PF_6_ to the reactional mixture. The compound was further purified *via* alumina column chromatography (MeCN:toluene 1:1 as eluent) and the final product isolated/air-dried by vacuum filtration following precipitation of the salt into Et_2_O. B) In this method, we followed a literature procedures (Arana & Abruña, 1993; Vogler *et al.*, 1996) by reacting tppz with 2 equiv of the mononuclear Ru^III^Cl_3_(tpy) complex for 24 h in refluxing EtOH/H_2_O (2:1 vol. mixture), under Ar and with excess Et_3_N added. The solid product was isolated and purified as described for method A. The identity of the cation [(tpy)Ru^II^(tppz)Ru^II^(tpy)]^4+^ (I) in solution was also confirmed by electrochemical and spectroscopic measurements. Single crystals suitable for X-ray analysis were obtained by slow diffusion of Et_2_O into concentrated MeCN solutions of [(tpy)Ru(tppz)Ru(tpy)](PF_6_)_4_.

### Refinement

Two PF_6_^-^ counterions and one MeCN solvent molecule were disordered, and each refined in two positions. The site-occupancy-factors (sof) of disordered pairs of atoms were refined and tied to sum to 1.0. The sof for the first anion (atoms P2, F7 to F12) was refined to 0.62 (2). The sof for the second anion (atoms P4, F19 to F24) was refined to 0.57 (2). The MeCN sof (atoms N13, C55, C56) was refined to 0.64 (1). For the first anion, only atoms F7, F9, F10, and F12 were disordered. Bond distances of disordered molecule pairs were restrained to have identical values. The disordered atoms P4 and P4' were constrained to have identical temperature factors. For the disordered MeCN molecule, atoms were restrained to have similar U_ij_ components. All H atoms were idealized and refined as riding atoms, with C-H = 0.93 Å (aromatic) or 0.96 Å (methyl) and U_iso_(H) = 1.2 (aromatic) or 1.5 (methyl) times U_eq_(C). Methyl torsion angles were refined from electron density.

### Crystal data

**Table:**

[Ru_2_(C_15_H_11_N_3_)_2_(C_24_H_16_N_6_)](PF_6_)_4_·4C_2_H_3_N	*F*(000) = 3592
*M**_r_* = 1801.20	*D*_x_ = 1.717 Mg m^−^^3^
Monoclinic, *P*2_1_/*c*	Mo *K*α radiation, λ = 0.71073 Å
Hall symbol: -P 2ybc	Cell parameters from 5749 reflections
*a* = 11.8871 (9) Å	θ = 4.4–44.8°
*b* = 31.824 (2) Å	µ = 0.64 mm^−^^1^
*c* = 18.5168 (14) Å	*T* = 120 K
β = 95.880 (1)°	Block, green
*V* = 6968.0 (9) Å^3^	0.18 × 0.10 × 0.08 mm
*Z* = 4	

### Data collection

**Table:**

Bruker D8 with APEXII CCD diffractometer	12753 independent reflections
Radiation source: fine-focus sealed tube	8864 reflections with *I* > 2σ(*I*)
Graphite monochromator	*R*_int_ = 0.107
ω scans	θ_max_ = 25.4°, θ_min_ = 1.8°
Absorption correction: multi-scan (*SADABS*; Sheldrick, 2008)	*h* = −14→14
*T*_min_ = 0.893, *T*_max_ = 0.950	*k* = −38→38
67490 measured reflections	*l* = −22→22

### Refinement

**Table:**

Refinement on *F*^2^	Primary atom site location: structure-invariant direct methods
Least-squares matrix: full	Secondary atom site location: difference Fourier map
*R*[*F*^2^ > 2σ(*F*^2^)] = 0.045	Hydrogen site location: inferred from neighbouring sites
*wR*(*F*^2^) = 0.102	H-atom parameters constrained
*S* = 1.10	*w* = 1/[σ^2^(*F*_o_^2^) + (0.0305*P*)^2^] where *P* = (*F*_o_^2^ + 2*F*_c_^2^)/3
12753 reflections	(Δ/σ)_max_ = 0.001
1101 parameters	Δρ_max_ = 0.75 e Å^−^^3^
78 restraints	Δρ_min_ = −0.50 e Å^−^^3^

### Special details

**Table:**

Geometry. All e.s.d.'s (except the e.s.d. in the dihedral angle between two l.s. planes) are estimated using the full covariance matrix. The cell e.s.d.'s are taken into account individually in the estimation of e.s.d.'s in distances, angles and torsion angles; correlations between e.s.d.'s in cell parameters are only used when they are defined by crystal symmetry. An approximate (isotropic) treatment of cell e.s.d.'s is used for estimating e.s.d.'s involving l.s. planes.
Refinement. Refinement of *F*^2^ against ALL reflections. The weighted *R*-factor *wR* and goodness of fit *S* are based on *F*^2^, conventional *R*-factors *R* are based on *F*, with *F* set to zero for negative *F*^2^. The threshold expression of *F*^2^ > σ(*F*^2^) is used only for calculating *R*-factors(gt) *etc*. and is not relevant to the choice of reflections for refinement. *R*-factors based on *F*^2^ are statistically about twice as large as those based on *F*, and *R*- factors based on ALL data will be even larger.

### Fractional atomic coordinates and isotropic or equivalent isotropic displacement parameters (Å^2^)

**Table:**

	*x*	*y*	*z*	*U*_iso_*/*U*_eq_	Occ. (<1)
Ru1	0.28004 (3)	0.035873 (10)	0.746568 (18)	0.01496 (9)	
Ru2	0.07869 (3)	0.229580 (10)	0.741853 (18)	0.01721 (10)	
P1	0.25514 (9)	0.90082 (4)	0.56200 (6)	0.0227 (3)	
P2	0.28371 (11)	0.44051 (4)	0.55316 (7)	0.0335 (3)	
P3	0.68786 (11)	0.30383 (4)	0.57181 (7)	0.0338 (3)	
F1	0.25658 (19)	0.90561 (7)	0.64831 (12)	0.0268 (6)	
F2	0.24448 (19)	0.85077 (7)	0.56958 (14)	0.0324 (6)	
F3	0.38934 (19)	0.89688 (8)	0.56969 (13)	0.0331 (6)	
F4	0.2513 (2)	0.89662 (8)	0.47551 (13)	0.0372 (7)	
F5	0.26664 (19)	0.95111 (7)	0.55527 (12)	0.0263 (6)	
F6	0.12030 (19)	0.90529 (7)	0.55462 (13)	0.0279 (6)	
F8	0.3444 (3)	0.39580 (9)	0.55286 (16)	0.0592 (9)	
F11	0.2215 (3)	0.48488 (10)	0.55481 (17)	0.0639 (10)	
F7	0.3737 (7)	0.4580 (2)	0.6111 (5)	0.061 (3)	0.620 (16)
F9	0.2061 (7)	0.42705 (18)	0.6146 (5)	0.052 (3)	0.620 (16)
F10	0.1800 (9)	0.4271 (2)	0.4950 (6)	0.097 (4)	0.620 (16)
F12	0.3450 (10)	0.4581 (3)	0.4896 (6)	0.077 (4)	0.620 (16)
F7'	0.2920 (19)	0.4335 (3)	0.6394 (4)	0.075 (7)	0.380 (16)
F9'	0.1765 (9)	0.4112 (5)	0.5478 (11)	0.098 (7)	0.380 (16)
F10'	0.2855 (15)	0.4379 (6)	0.4691 (5)	0.071 (6)	0.380 (16)
F12'	0.4061 (9)	0.4613 (3)	0.5608 (13)	0.086 (7)	0.380 (16)
F13	0.6356 (2)	0.31570 (8)	0.64503 (16)	0.0503 (8)	
F14	0.6916 (4)	0.25661 (10)	0.5933 (2)	0.1006 (15)	
F15	0.8113 (2)	0.31061 (10)	0.61355 (15)	0.0609 (9)	
F16	0.7432 (3)	0.29210 (10)	0.50021 (15)	0.0635 (9)	
F17	0.6918 (3)	0.35241 (9)	0.55080 (15)	0.0578 (9)	
F18	0.5676 (3)	0.29980 (11)	0.5294 (2)	0.0859 (12)	
P4	0.2383 (7)	0.1122 (3)	0.4097 (5)	0.0257 (6)	0.57 (2)
F19	0.3620 (9)	0.0939 (5)	0.4252 (8)	0.058 (4)	0.57 (2)
F20	0.1908 (10)	0.0826 (3)	0.4676 (3)	0.048 (3)	0.57 (2)
F21	0.2668 (14)	0.1450 (4)	0.4736 (7)	0.054 (3)	0.57 (2)
F22	0.1166 (7)	0.1327 (5)	0.3952 (5)	0.051 (3)	0.57 (2)
F23	0.2835 (11)	0.1430 (4)	0.3528 (6)	0.068 (3)	0.57 (2)
F24	0.2076 (12)	0.0789 (4)	0.3475 (7)	0.069 (4)	0.57 (2)
P4'	0.2409 (10)	0.1081 (4)	0.4068 (7)	0.0257 (6)	0.43 (2)
F19'	0.3744 (9)	0.1053 (5)	0.4070 (9)	0.038 (4)	0.43 (2)
F20'	0.2414 (12)	0.0637 (5)	0.4463 (10)	0.068 (6)	0.43 (2)
F21'	0.2594 (15)	0.1316 (8)	0.4826 (9)	0.076 (7)	0.43 (2)
F22'	0.1071 (8)	0.1098 (5)	0.4053 (7)	0.040 (3)	0.43 (2)
F23'	0.2421 (14)	0.1526 (3)	0.3683 (11)	0.065 (5)	0.43 (2)
F24'	0.2257 (12)	0.0861 (5)	0.3299 (8)	0.042 (4)	0.43 (2)
N1	0.3936 (3)	0.03905 (10)	0.66882 (18)	0.0181 (8)	
N2	0.3472 (3)	−0.02106 (10)	0.75424 (17)	0.0173 (8)	
N3	0.1865 (3)	0.00855 (10)	0.82253 (17)	0.0193 (8)	
N4	0.1428 (3)	0.02977 (10)	0.66999 (17)	0.0167 (8)	
N5	0.2211 (3)	0.09345 (10)	0.74346 (17)	0.0149 (7)	
N6	0.3934 (3)	0.06344 (10)	0.82402 (17)	0.0163 (8)	
N7	0.0196 (3)	0.21001 (10)	0.63882 (17)	0.0167 (8)	
N8	0.1398 (3)	0.17199 (10)	0.74223 (17)	0.0160 (8)	
N9	0.1634 (3)	0.22700 (10)	0.84401 (17)	0.0175 (8)	
N10	0.2107 (3)	0.26278 (10)	0.70475 (17)	0.0204 (8)	
N11	0.0202 (3)	0.28805 (10)	0.74401 (17)	0.0196 (8)	
N12	−0.0772 (3)	0.21994 (10)	0.78029 (18)	0.0209 (8)	
N15	0.4640 (5)	0.16296 (15)	0.6902 (3)	0.0695 (16)	
N16	0.8158 (7)	0.08326 (18)	0.7468 (3)	0.110 (3)	
C1	0.4069 (4)	0.07013 (14)	0.6217 (2)	0.0265 (11)	
H1	0.3620	0.0940	0.6231	0.032*	
C2	0.4843 (4)	0.06829 (16)	0.5711 (2)	0.0349 (12)	
H2	0.4913	0.0905	0.5393	0.042*	
C3	0.5510 (4)	0.03303 (16)	0.5685 (3)	0.0358 (12)	
H3	0.6050	0.0314	0.5357	0.043*	
C4	0.5367 (4)	0.00044 (15)	0.6148 (2)	0.0283 (11)	
H4	0.5801	−0.0238	0.6129	0.034*	
C5	0.4577 (3)	0.00342 (13)	0.6645 (2)	0.0218 (10)	
C6	0.4335 (3)	−0.03055 (13)	0.7148 (2)	0.0229 (10)	
C7	0.4878 (4)	−0.06887 (14)	0.7234 (3)	0.0324 (12)	
H7	0.5473	−0.0756	0.6966	0.039*	
C8	0.4517 (4)	−0.09672 (15)	0.7725 (3)	0.0380 (13)	
H8	0.4868	−0.1228	0.7785	0.046*	
C9	0.3652 (4)	−0.08701 (14)	0.8130 (3)	0.0360 (13)	
H9	0.3420	−0.1060	0.8466	0.043*	
C10	0.3126 (4)	−0.04804 (13)	0.8030 (2)	0.0239 (10)	
C11	0.2183 (4)	−0.03205 (13)	0.8403 (2)	0.0238 (10)	
C12	0.1585 (4)	−0.05518 (15)	0.8870 (3)	0.0382 (13)	
H12	0.1801	−0.0826	0.8990	0.046*	
C13	0.0672 (4)	−0.03753 (16)	0.9158 (3)	0.0418 (14)	
H13	0.0270	−0.0530	0.9472	0.050*	
C14	0.0355 (4)	0.00323 (16)	0.8978 (2)	0.0356 (12)	
H14	−0.0265	0.0156	0.9162	0.043*	
C15	0.0984 (4)	0.02503 (14)	0.8517 (2)	0.0264 (11)	
H15	0.0785	0.0527	0.8404	0.032*	
C16	0.0985 (3)	−0.00642 (12)	0.6426 (2)	0.0186 (10)	
H16	0.1383	−0.0312	0.6527	0.022*	
C17	−0.0039 (3)	−0.00814 (13)	0.6002 (2)	0.0217 (10)	
H17	−0.0318	−0.0337	0.5818	0.026*	
C18	−0.0641 (3)	0.02809 (13)	0.5853 (2)	0.0220 (10)	
H18	−0.1333	0.0275	0.5569	0.026*	
C19	−0.0198 (3)	0.06579 (13)	0.6135 (2)	0.0183 (9)	
H19	−0.0606	0.0906	0.6052	0.022*	
C20	0.0844 (3)	0.06646 (12)	0.6537 (2)	0.0166 (9)	
C21	0.1359 (3)	0.10381 (12)	0.6913 (2)	0.0158 (9)	
C22	0.2681 (3)	0.12141 (12)	0.7936 (2)	0.0151 (9)	
C23	0.3740 (3)	0.10548 (13)	0.8336 (2)	0.0186 (9)	
C24	0.4533 (3)	0.13076 (13)	0.8722 (2)	0.0197 (10)	
H24	0.4414	0.1595	0.8756	0.024*	
C25	0.5505 (3)	0.11264 (13)	0.9058 (2)	0.0244 (10)	
H25	0.6049	0.1292	0.9319	0.029*	
C26	0.5665 (4)	0.07012 (13)	0.9005 (2)	0.0242 (10)	
H26	0.6296	0.0573	0.9249	0.029*	
C27	0.4869 (3)	0.04663 (13)	0.8580 (2)	0.0211 (10)	
H27	0.4992	0.0180	0.8529	0.025*	
C28	−0.0338 (3)	0.23341 (13)	0.5858 (2)	0.0207 (10)	
H28	−0.0563	0.2605	0.5969	0.025*	
C29	−0.0568 (3)	0.21909 (12)	0.5156 (2)	0.0191 (10)	
H29	−0.0971	0.2357	0.4806	0.023*	
C30	−0.0194 (3)	0.17995 (13)	0.4977 (2)	0.0203 (10)	
H30	−0.0301	0.1704	0.4501	0.024*	
C31	0.0343 (3)	0.15503 (12)	0.5520 (2)	0.0172 (9)	
H31	0.0605	0.1285	0.5408	0.021*	
C32	0.0491 (3)	0.16933 (12)	0.6225 (2)	0.0166 (9)	
C33	0.1078 (3)	0.14650 (12)	0.6842 (2)	0.0152 (9)	
C34	0.2115 (3)	0.15964 (12)	0.7997 (2)	0.0158 (9)	
C35	0.2151 (3)	0.18920 (12)	0.8611 (2)	0.0174 (9)	
C36	0.2575 (3)	0.18020 (13)	0.9310 (2)	0.0233 (10)	
H36	0.2867	0.1537	0.9426	0.028*	
C37	0.2570 (4)	0.21042 (14)	0.9842 (2)	0.0269 (11)	
H37	0.2862	0.2045	1.0316	0.032*	
C38	0.2123 (3)	0.24982 (14)	0.9662 (2)	0.0253 (10)	
H38	0.2148	0.2712	1.0006	0.030*	
C39	0.1646 (3)	0.25648 (13)	0.8968 (2)	0.0222 (10)	
H39	0.1315	0.2824	0.8853	0.027*	
C40	0.3096 (4)	0.24702 (13)	0.6877 (2)	0.0232 (10)	
H40	0.3215	0.2182	0.6911	0.028*	
C41	0.3941 (4)	0.27222 (13)	0.6653 (2)	0.0256 (10)	
H41	0.4623	0.2606	0.6547	0.031*	
C42	0.3758 (4)	0.31444 (14)	0.6589 (2)	0.0292 (11)	
H42	0.4306	0.3317	0.6421	0.035*	
C43	0.2758 (4)	0.33143 (14)	0.6775 (2)	0.0281 (11)	
H43	0.2639	0.3603	0.6746	0.034*	
C44	0.1933 (4)	0.30535 (13)	0.7005 (2)	0.0219 (10)	
C45	0.0855 (4)	0.31972 (13)	0.7234 (2)	0.0243 (11)	
C46	0.0477 (4)	0.36090 (13)	0.7270 (2)	0.0314 (12)	
H46	0.0910	0.3830	0.7121	0.038*	
C47	−0.0548 (4)	0.36862 (14)	0.7531 (2)	0.0352 (12)	
H47	−0.0805	0.3961	0.7560	0.042*	
C48	−0.1195 (4)	0.33595 (14)	0.7749 (2)	0.0319 (12)	
H48	−0.1884	0.3412	0.7929	0.038*	
C49	−0.0805 (4)	0.29505 (13)	0.7697 (2)	0.0227 (10)	
C50	−0.1377 (4)	0.25614 (14)	0.7883 (2)	0.0245 (11)	
C51	−0.2441 (4)	0.25438 (16)	0.8121 (2)	0.0337 (12)	
H51	−0.2842	0.2790	0.8178	0.040*	
C52	−0.2905 (4)	0.21650 (17)	0.8274 (3)	0.0445 (14)	
H52	−0.3622	0.2153	0.8433	0.053*	
C53	−0.2303 (4)	0.18012 (17)	0.8190 (3)	0.0422 (13)	
H53	−0.2603	0.1541	0.8294	0.051*	
C54	−0.1243 (4)	0.18309 (14)	0.7947 (2)	0.0292 (11)	
H54	−0.0842	0.1585	0.7881	0.035*	
N13	0.0164 (8)	0.1179 (3)	0.8910 (5)	0.068 (3)	0.639 (11)
C55	0.0032 (12)	0.1310 (3)	0.9445 (7)	0.056 (3)	0.639 (11)
C56	0.006 (2)	0.1500 (7)	1.0178 (13)	0.062 (6)	0.639 (11)
H56A	0.0421	0.1769	1.0180	0.094*	0.639 (11)
H56B	0.0467	0.1320	1.0527	0.094*	0.639 (11)
H56C	−0.0703	0.1534	1.0302	0.094*	0.639 (11)
C57	0.4656 (4)	0.26977 (16)	0.8956 (3)	0.0340 (12)	
N13'	−0.112 (2)	0.0966 (5)	0.9245 (9)	0.112 (10)	0.361 (11)
C55'	−0.070 (3)	0.1201 (6)	0.9621 (10)	0.070 (7)	0.361 (11)
C56'	−0.010 (5)	0.1545 (15)	1.003 (3)	0.089 (15)	0.361 (11)
H56D	0.0695	0.1494	1.0074	0.133*	0.361 (11)
H56E	−0.0357	0.1561	1.0507	0.133*	0.361 (11)
H56F	−0.0265	0.1806	0.9781	0.133*	0.361 (11)
N14	0.4391 (3)	0.24561 (14)	0.8530 (2)	0.0425 (11)	
C58	0.5019 (4)	0.30040 (16)	0.9534 (3)	0.0525 (15)	
H58A	0.5589	0.2879	0.9872	0.079*	
H58B	0.4382	0.3083	0.9783	0.079*	
H58C	0.5323	0.3249	0.9321	0.079*	
C59	0.5485 (6)	0.16313 (16)	0.6670 (3)	0.0489 (16)	
C60	0.6584 (5)	0.16184 (17)	0.6398 (3)	0.0640 (18)	
H60A	0.6938	0.1889	0.6459	0.096*	
H60B	0.6495	0.1546	0.5892	0.096*	
H60C	0.7048	0.1412	0.6663	0.096*	
C61	0.7993 (5)	0.0490 (2)	0.7523 (3)	0.0512 (15)	
C62	0.7804 (5)	0.00479 (17)	0.7588 (3)	0.0589 (17)	
H62A	0.7081	0.0001	0.7762	0.088*	
H62B	0.8387	−0.0072	0.7923	0.088*	
H62C	0.7817	−0.0083	0.7121	0.088*	

### Atomic displacement parameters (Å^2^)

**Table:**

	*U*^11^	*U*^22^	*U*^33^	*U*^12^	*U*^13^	*U*^23^
Ru1	0.01606 (19)	0.01215 (18)	0.01674 (19)	0.00127 (14)	0.00196 (14)	−0.00011 (14)
Ru2	0.0201 (2)	0.01344 (19)	0.01807 (19)	0.00433 (15)	0.00167 (15)	−0.00166 (15)
P1	0.0194 (6)	0.0221 (7)	0.0266 (7)	0.0011 (5)	0.0021 (5)	−0.0013 (5)
P2	0.0376 (8)	0.0355 (8)	0.0278 (7)	−0.0062 (6)	0.0048 (6)	0.0005 (6)
P3	0.0380 (8)	0.0253 (7)	0.0379 (8)	0.0073 (6)	0.0027 (6)	−0.0078 (6)
F1	0.0291 (15)	0.0270 (14)	0.0245 (14)	−0.0054 (11)	0.0043 (11)	0.0011 (11)
F2	0.0264 (15)	0.0183 (14)	0.0521 (18)	0.0015 (11)	0.0018 (13)	−0.0053 (12)
F3	0.0191 (14)	0.0351 (16)	0.0455 (17)	0.0015 (12)	0.0057 (12)	−0.0005 (13)
F4	0.0368 (17)	0.0472 (18)	0.0269 (15)	0.0078 (13)	0.0008 (12)	−0.0110 (13)
F5	0.0304 (15)	0.0200 (13)	0.0294 (15)	−0.0019 (11)	0.0066 (12)	0.0039 (11)
F6	0.0183 (13)	0.0254 (14)	0.0398 (16)	−0.0003 (11)	0.0014 (11)	−0.0067 (12)
F8	0.094 (3)	0.0336 (18)	0.053 (2)	0.0041 (17)	0.0224 (18)	−0.0046 (15)
F11	0.068 (2)	0.062 (2)	0.066 (2)	0.0283 (18)	0.0262 (18)	0.0213 (18)
F7	0.046 (5)	0.038 (4)	0.094 (7)	−0.010 (3)	−0.025 (4)	−0.018 (4)
F9	0.056 (5)	0.038 (3)	0.068 (6)	0.007 (3)	0.030 (4)	0.025 (3)
F10	0.115 (7)	0.081 (5)	0.080 (7)	−0.034 (5)	−0.055 (6)	0.024 (4)
F12	0.121 (9)	0.053 (5)	0.070 (7)	0.012 (5)	0.071 (6)	0.015 (4)
F7'	0.16 (2)	0.046 (7)	0.019 (5)	0.048 (10)	0.006 (7)	−0.006 (4)
F9'	0.033 (6)	0.127 (12)	0.138 (18)	−0.025 (7)	0.028 (8)	−0.018 (11)
F10'	0.112 (14)	0.074 (11)	0.025 (5)	0.055 (9)	−0.003 (7)	0.002 (6)
F12'	0.046 (7)	0.043 (6)	0.17 (2)	−0.023 (5)	0.021 (9)	−0.016 (10)
F13	0.0499 (19)	0.0447 (18)	0.061 (2)	−0.0099 (15)	0.0293 (16)	−0.0184 (15)
F14	0.204 (5)	0.0232 (19)	0.080 (3)	0.005 (2)	0.039 (3)	0.0015 (18)
F15	0.0428 (19)	0.095 (3)	0.0421 (19)	0.0274 (18)	−0.0063 (15)	−0.0161 (18)
F16	0.085 (3)	0.069 (2)	0.0383 (19)	0.0147 (19)	0.0131 (17)	−0.0218 (17)
F17	0.096 (3)	0.0345 (18)	0.0394 (18)	0.0012 (17)	−0.0082 (17)	−0.0002 (14)
F18	0.048 (2)	0.100 (3)	0.105 (3)	−0.004 (2)	−0.017 (2)	−0.047 (2)
P4	0.0226 (7)	0.0271 (16)	0.0282 (9)	0.0022 (9)	0.0056 (6)	−0.0040 (10)
F19	0.033 (5)	0.098 (9)	0.042 (6)	0.047 (5)	0.001 (4)	0.001 (5)
F20	0.062 (6)	0.030 (5)	0.053 (4)	−0.010 (4)	0.007 (3)	0.009 (3)
F21	0.056 (6)	0.044 (5)	0.062 (6)	−0.016 (4)	0.014 (4)	−0.029 (4)
F22	0.028 (4)	0.081 (8)	0.044 (4)	0.024 (5)	0.002 (3)	−0.011 (5)
F23	0.065 (7)	0.086 (8)	0.054 (5)	−0.004 (5)	0.018 (5)	0.029 (5)
F24	0.093 (8)	0.071 (7)	0.046 (6)	−0.024 (5)	0.013 (5)	−0.041 (5)
P4'	0.0226 (7)	0.0271 (16)	0.0282 (9)	0.0022 (9)	0.0056 (6)	−0.0040 (10)
F19'	0.024 (5)	0.043 (6)	0.046 (9)	−0.006 (4)	0.000 (4)	−0.015 (6)
F20'	0.046 (7)	0.039 (8)	0.114 (10)	−0.012 (6)	−0.022 (6)	0.045 (7)
F21'	0.031 (8)	0.143 (18)	0.052 (8)	0.022 (9)	−0.001 (6)	−0.057 (10)
F22'	0.022 (4)	0.038 (7)	0.062 (6)	−0.003 (4)	0.005 (4)	−0.010 (5)
F23'	0.060 (10)	0.020 (5)	0.107 (12)	−0.002 (5)	−0.029 (7)	0.022 (6)
F24'	0.030 (6)	0.056 (7)	0.038 (7)	0.025 (6)	−0.009 (5)	−0.019 (5)
N1	0.0149 (19)	0.0183 (19)	0.021 (2)	−0.0022 (15)	0.0005 (15)	−0.0010 (16)
N2	0.019 (2)	0.0140 (18)	0.0180 (19)	0.0019 (15)	−0.0035 (15)	−0.0024 (15)
N3	0.021 (2)	0.019 (2)	0.0173 (19)	−0.0043 (16)	−0.0003 (16)	−0.0003 (16)
N4	0.0180 (19)	0.0160 (19)	0.0169 (19)	0.0018 (15)	0.0056 (15)	0.0002 (15)
N5	0.0135 (18)	0.0169 (19)	0.0142 (18)	−0.0005 (15)	0.0012 (15)	0.0003 (15)
N6	0.0180 (19)	0.0124 (18)	0.0190 (19)	−0.0018 (15)	0.0050 (15)	−0.0016 (15)
N7	0.0164 (19)	0.0172 (19)	0.0165 (19)	0.0029 (15)	0.0022 (15)	0.0014 (15)
N8	0.0193 (19)	0.0136 (18)	0.0152 (19)	0.0009 (15)	0.0030 (15)	−0.0003 (15)
N9	0.020 (2)	0.0138 (19)	0.0193 (19)	−0.0008 (15)	0.0035 (15)	−0.0021 (15)
N10	0.024 (2)	0.019 (2)	0.018 (2)	0.0008 (16)	−0.0003 (16)	−0.0025 (15)
N11	0.026 (2)	0.0165 (19)	0.0148 (19)	0.0041 (16)	−0.0021 (16)	−0.0067 (15)
N12	0.023 (2)	0.022 (2)	0.019 (2)	0.0052 (16)	0.0040 (16)	−0.0026 (16)
N15	0.086 (4)	0.046 (3)	0.078 (4)	0.018 (3)	0.016 (3)	−0.013 (3)
N16	0.216 (8)	0.040 (4)	0.078 (5)	0.014 (4)	0.030 (5)	−0.010 (3)
C1	0.029 (3)	0.024 (3)	0.026 (3)	−0.003 (2)	0.001 (2)	0.004 (2)
C2	0.042 (3)	0.043 (3)	0.022 (3)	−0.015 (3)	0.010 (2)	0.002 (2)
C3	0.027 (3)	0.048 (3)	0.035 (3)	−0.014 (3)	0.013 (2)	−0.010 (3)
C4	0.023 (3)	0.034 (3)	0.029 (3)	−0.003 (2)	0.005 (2)	−0.011 (2)
C5	0.016 (2)	0.024 (3)	0.025 (3)	0.0004 (19)	0.0007 (19)	−0.007 (2)
C6	0.021 (2)	0.021 (2)	0.025 (3)	0.003 (2)	−0.005 (2)	−0.007 (2)
C7	0.030 (3)	0.031 (3)	0.034 (3)	0.015 (2)	−0.013 (2)	−0.006 (2)
C8	0.046 (3)	0.021 (3)	0.043 (3)	0.017 (2)	−0.015 (3)	−0.001 (2)
C9	0.052 (3)	0.018 (3)	0.036 (3)	0.002 (2)	−0.006 (3)	0.009 (2)
C10	0.036 (3)	0.013 (2)	0.021 (3)	−0.003 (2)	−0.002 (2)	0.0008 (19)
C11	0.032 (3)	0.020 (2)	0.019 (2)	−0.005 (2)	0.000 (2)	0.001 (2)
C12	0.053 (4)	0.029 (3)	0.032 (3)	−0.016 (3)	0.005 (3)	0.006 (2)
C13	0.057 (4)	0.040 (3)	0.030 (3)	−0.025 (3)	0.016 (3)	0.000 (3)
C14	0.034 (3)	0.046 (3)	0.029 (3)	−0.009 (3)	0.013 (2)	−0.006 (2)
C15	0.030 (3)	0.026 (3)	0.024 (3)	−0.007 (2)	0.004 (2)	−0.007 (2)
C16	0.022 (2)	0.010 (2)	0.023 (2)	0.0006 (18)	0.0027 (19)	−0.0040 (18)
C17	0.027 (3)	0.015 (2)	0.023 (2)	−0.0052 (19)	0.004 (2)	−0.0007 (19)
C18	0.020 (2)	0.024 (3)	0.022 (2)	−0.005 (2)	−0.0007 (19)	0.000 (2)
C19	0.017 (2)	0.018 (2)	0.020 (2)	0.0017 (18)	0.0021 (19)	0.0007 (18)
C20	0.018 (2)	0.015 (2)	0.016 (2)	0.0019 (18)	0.0028 (18)	0.0000 (18)
C21	0.019 (2)	0.016 (2)	0.014 (2)	−0.0002 (18)	0.0062 (18)	0.0001 (18)
C22	0.020 (2)	0.013 (2)	0.014 (2)	−0.0004 (17)	0.0060 (18)	0.0008 (17)
C23	0.019 (2)	0.020 (2)	0.018 (2)	−0.0009 (19)	0.0044 (18)	0.0010 (19)
C24	0.023 (2)	0.016 (2)	0.019 (2)	0.0019 (19)	−0.0014 (19)	−0.0029 (18)
C25	0.020 (2)	0.029 (3)	0.024 (3)	−0.004 (2)	−0.002 (2)	−0.004 (2)
C26	0.023 (3)	0.026 (3)	0.021 (2)	0.004 (2)	−0.005 (2)	−0.005 (2)
C27	0.025 (3)	0.018 (2)	0.020 (2)	0.0075 (19)	0.003 (2)	0.0008 (19)
C28	0.021 (2)	0.017 (2)	0.024 (3)	0.0027 (19)	0.0024 (19)	0.0024 (19)
C29	0.019 (2)	0.019 (2)	0.018 (2)	0.0007 (18)	−0.0043 (18)	0.0033 (18)
C30	0.022 (2)	0.023 (2)	0.016 (2)	−0.0064 (19)	0.0024 (19)	−0.0014 (19)
C31	0.018 (2)	0.012 (2)	0.022 (2)	0.0019 (17)	0.0026 (18)	−0.0024 (18)
C32	0.016 (2)	0.017 (2)	0.017 (2)	0.0008 (18)	0.0026 (18)	−0.0034 (18)
C33	0.017 (2)	0.015 (2)	0.014 (2)	−0.0019 (17)	0.0043 (18)	0.0000 (18)
C34	0.017 (2)	0.014 (2)	0.017 (2)	−0.0034 (18)	0.0050 (18)	0.0020 (18)
C35	0.018 (2)	0.016 (2)	0.019 (2)	0.0001 (18)	0.0052 (18)	0.0004 (18)
C36	0.029 (3)	0.020 (2)	0.020 (2)	0.004 (2)	−0.001 (2)	0.000 (2)
C37	0.033 (3)	0.030 (3)	0.016 (2)	0.004 (2)	−0.001 (2)	0.002 (2)
C38	0.029 (3)	0.025 (3)	0.022 (3)	0.000 (2)	0.002 (2)	−0.006 (2)
C39	0.025 (3)	0.016 (2)	0.025 (3)	0.0071 (19)	0.002 (2)	−0.004 (2)
C40	0.026 (3)	0.020 (2)	0.023 (2)	0.000 (2)	−0.003 (2)	0.0002 (19)
C41	0.023 (3)	0.029 (3)	0.024 (3)	−0.004 (2)	0.000 (2)	0.001 (2)
C42	0.032 (3)	0.032 (3)	0.023 (3)	−0.014 (2)	−0.001 (2)	−0.005 (2)
C43	0.046 (3)	0.018 (2)	0.019 (3)	−0.009 (2)	−0.001 (2)	−0.003 (2)
C44	0.033 (3)	0.017 (2)	0.014 (2)	0.002 (2)	−0.0072 (19)	−0.0017 (19)
C45	0.034 (3)	0.014 (2)	0.022 (3)	0.006 (2)	−0.008 (2)	−0.0020 (19)
C46	0.047 (3)	0.014 (2)	0.031 (3)	0.003 (2)	−0.009 (2)	0.000 (2)
C47	0.048 (3)	0.015 (3)	0.040 (3)	0.012 (2)	−0.007 (3)	−0.004 (2)
C48	0.035 (3)	0.032 (3)	0.028 (3)	0.018 (2)	−0.005 (2)	−0.011 (2)
C49	0.030 (3)	0.023 (3)	0.016 (2)	0.012 (2)	0.001 (2)	−0.0050 (19)
C50	0.025 (3)	0.035 (3)	0.013 (2)	0.010 (2)	−0.0009 (19)	−0.007 (2)
C51	0.027 (3)	0.043 (3)	0.031 (3)	0.015 (2)	0.003 (2)	−0.006 (2)
C52	0.032 (3)	0.050 (4)	0.055 (4)	0.005 (3)	0.022 (3)	−0.003 (3)
C53	0.032 (3)	0.045 (3)	0.052 (4)	−0.002 (3)	0.018 (3)	0.002 (3)
C54	0.030 (3)	0.027 (3)	0.033 (3)	0.005 (2)	0.010 (2)	0.000 (2)
N13	0.080 (7)	0.039 (5)	0.082 (7)	0.021 (5)	0.000 (6)	−0.004 (5)
C55	0.066 (9)	0.032 (6)	0.069 (9)	0.016 (6)	0.012 (6)	−0.001 (6)
C56	0.092 (17)	0.027 (7)	0.077 (11)	−0.002 (8)	0.051 (10)	−0.009 (7)
C57	0.020 (3)	0.036 (3)	0.046 (3)	0.007 (2)	0.004 (2)	0.006 (3)
N13'	0.23 (3)	0.056 (12)	0.038 (10)	0.041 (13)	−0.055 (13)	−0.011 (8)
C55'	0.118 (19)	0.050 (13)	0.046 (12)	0.039 (13)	0.025 (14)	0.018 (10)
C56'	0.054 (18)	0.10 (3)	0.11 (3)	0.00 (2)	0.011 (17)	0.02 (2)
N14	0.033 (3)	0.048 (3)	0.047 (3)	0.004 (2)	0.005 (2)	−0.003 (2)
C58	0.042 (3)	0.034 (3)	0.079 (4)	−0.001 (3)	−0.006 (3)	−0.010 (3)
C59	0.077 (5)	0.030 (3)	0.041 (4)	0.014 (3)	0.012 (3)	−0.009 (3)
C60	0.107 (6)	0.044 (4)	0.043 (4)	0.014 (4)	0.018 (4)	−0.001 (3)
C61	0.073 (4)	0.047 (4)	0.036 (3)	0.009 (3)	0.015 (3)	−0.003 (3)
C62	0.066 (4)	0.048 (4)	0.059 (4)	−0.015 (3)	−0.012 (3)	0.019 (3)

### Geometric parameters (Å, º)

**Table:**

Ru1—N5	1.960 (3)	C13—C14	1.382 (6)
Ru1—N2	1.979 (3)	C13—H13	0.9300
Ru1—N4	2.058 (3)	C14—C15	1.378 (6)
Ru1—N6	2.060 (3)	C14—H14	0.9300
Ru1—N3	2.071 (3)	C15—H15	0.9300
Ru1—N1	2.074 (3)	C16—C17	1.381 (5)
Ru2—N8	1.971 (3)	C16—H16	0.9300
Ru2—N11	1.989 (3)	C17—C18	1.370 (5)
Ru2—N9	2.051 (3)	C17—H17	0.9300
Ru2—N7	2.061 (3)	C18—C19	1.390 (5)
Ru2—N10	2.067 (3)	C18—H18	0.9300
Ru2—N12	2.074 (3)	C19—C20	1.379 (5)
P1—F3	1.592 (2)	C19—H19	0.9300
P1—F6	1.601 (2)	C20—C21	1.478 (5)
P1—F4	1.603 (3)	C21—C33	1.402 (5)
P1—F1	1.604 (2)	C22—C34	1.401 (5)
P1—F2	1.605 (2)	C22—C23	1.483 (5)
P1—F5	1.612 (2)	C23—C24	1.382 (5)
P2—F7	1.539 (6)	C24—C25	1.381 (5)
P2—F12	1.550 (6)	C24—H24	0.9300
P2—F10'	1.560 (10)	C25—C26	1.371 (6)
P2—F9'	1.574 (10)	C25—H25	0.9300
P2—F12'	1.590 (10)	C26—C27	1.387 (6)
P2—F9	1.595 (5)	C26—H26	0.9300
P2—F11	1.595 (3)	C27—H27	0.9300
P2—F8	1.596 (3)	C28—C29	1.379 (5)
P2—F7'	1.605 (8)	C28—H28	0.9300
P2—F10	1.608 (6)	C29—C30	1.374 (5)
P3—F14	1.554 (3)	C29—H29	0.9300
P3—F18	1.564 (3)	C30—C31	1.384 (5)
P3—F16	1.584 (3)	C30—H30	0.9300
P3—F13	1.594 (3)	C31—C32	1.376 (5)
P3—F17	1.596 (3)	C31—H31	0.9300
P3—F15	1.602 (3)	C32—C33	1.468 (5)
P4—F23	1.573 (9)	C34—C35	1.473 (5)
P4—F20	1.576 (8)	C35—C36	1.370 (5)
P4—F19	1.580 (8)	C36—C37	1.377 (6)
P4—F24	1.581 (9)	C36—H36	0.9300
P4—F22	1.584 (8)	C37—C38	1.389 (6)
P4—F21	1.587 (9)	C37—H37	0.9300
P4'—F24'	1.582 (11)	C38—C39	1.367 (5)
P4'—F21'	1.585 (11)	C38—H38	0.9300
P4'—F23'	1.587 (11)	C39—H39	0.9300
P4'—F19'	1.589 (11)	C40—C41	1.382 (6)
P4'—F22'	1.589 (11)	C40—H40	0.9300
P4'—F20'	1.590 (11)	C41—C42	1.364 (6)
N1—C1	1.339 (5)	C41—H41	0.9300
N1—C5	1.373 (5)	C42—C43	1.382 (6)
N2—C10	1.341 (5)	C42—H42	0.9300
N2—C6	1.352 (5)	C43—C44	1.384 (6)
N3—C15	1.334 (5)	C43—H43	0.9300
N3—C11	1.376 (5)	C44—C45	1.464 (6)
N4—C16	1.343 (5)	C45—C46	1.390 (5)
N4—C20	1.376 (5)	C46—C47	1.377 (6)
N5—C22	1.364 (5)	C46—H46	0.9300
N5—C21	1.366 (5)	C47—C48	1.378 (6)
N6—C27	1.332 (5)	C47—H47	0.9300
N6—C23	1.372 (5)	C48—C49	1.388 (5)
N7—C28	1.339 (5)	C48—H48	0.9300
N7—C32	1.383 (5)	C49—C50	1.470 (6)
N8—C34	1.352 (5)	C50—C51	1.382 (6)
N8—C33	1.369 (5)	C51—C52	1.367 (6)
N9—C39	1.354 (5)	C51—H51	0.9300
N9—C35	1.373 (5)	C52—C53	1.378 (6)
N10—C40	1.345 (5)	C52—H52	0.9300
N10—C44	1.371 (5)	C53—C54	1.384 (6)
N11—C49	1.351 (5)	C53—H53	0.9300
N11—C45	1.351 (5)	C54—H54	0.9300
N12—C54	1.338 (5)	N13—C55	1.100 (10)
N12—C50	1.374 (5)	C55—C56	1.484 (18)
N15—C59	1.132 (7)	C56—H56A	0.9600
N16—C61	1.114 (7)	C56—H56B	0.9600
C1—C2	1.379 (6)	C56—H56C	0.9600
C1—H1	0.9300	C57—N14	1.124 (6)
C2—C3	1.378 (6)	C57—C58	1.478 (7)
C2—H2	0.9300	N13'—C55'	1.105 (16)
C3—C4	1.367 (6)	C55'—C56'	1.47 (2)
C3—H3	0.9300	C56'—H56D	0.9600
C4—C5	1.384 (5)	C56'—H56E	0.9600
C4—H4	0.9300	C56'—H56F	0.9600
C5—C6	1.474 (6)	C58—H58A	0.9600
C6—C7	1.382 (6)	C58—H58B	0.9600
C7—C8	1.370 (6)	C58—H58C	0.9600
C7—H7	0.9300	C59—C60	1.449 (8)
C8—C9	1.369 (6)	C60—H60A	0.9600
C8—H8	0.9300	C60—H60B	0.9600
C9—C10	1.393 (6)	C60—H60C	0.9600
C9—H9	0.9300	C61—C62	1.432 (8)
C10—C11	1.467 (6)	C62—H62A	0.9600
C11—C12	1.386 (6)	C62—H62B	0.9600
C12—C13	1.377 (7)	C62—H62C	0.9600
C12—H12	0.9300		
			
N5—Ru1—N2	176.41 (13)	C4—C5—C6	124.0 (4)
N5—Ru1—N4	79.19 (13)	N2—C6—C7	120.4 (4)
N2—Ru1—N4	104.39 (13)	N2—C6—C5	112.7 (3)
N5—Ru1—N6	80.12 (13)	C7—C6—C5	126.9 (4)
N2—Ru1—N6	96.29 (13)	C8—C7—C6	118.3 (5)
N4—Ru1—N6	159.28 (12)	C8—C7—H7	120.9
N5—Ru1—N3	101.38 (13)	C6—C7—H7	120.9
N2—Ru1—N3	78.91 (13)	C9—C8—C7	121.4 (4)
N4—Ru1—N3	89.18 (12)	C9—C8—H8	119.3
N6—Ru1—N3	93.68 (12)	C7—C8—H8	119.3
N5—Ru1—N1	100.95 (13)	C8—C9—C10	118.7 (4)
N2—Ru1—N1	78.93 (13)	C8—C9—H9	120.7
N4—Ru1—N1	93.01 (12)	C10—C9—H9	120.7
N6—Ru1—N1	92.11 (12)	N2—C10—C9	119.7 (4)
N3—Ru1—N1	157.57 (13)	N2—C10—C11	113.4 (4)
N8—Ru2—N11	178.32 (14)	C9—C10—C11	126.9 (4)
N8—Ru2—N9	79.26 (13)	N3—C11—C12	120.1 (4)
N11—Ru2—N9	99.13 (13)	N3—C11—C10	114.7 (4)
N8—Ru2—N7	79.21 (13)	C12—C11—C10	125.1 (4)
N11—Ru2—N7	102.40 (13)	C13—C12—C11	120.0 (5)
N9—Ru2—N7	158.45 (13)	C13—C12—H12	120.0
N8—Ru2—N10	100.63 (13)	C11—C12—H12	120.0
N11—Ru2—N10	78.89 (14)	C12—C13—C14	119.7 (4)
N9—Ru2—N10	90.55 (13)	C12—C13—H13	120.1
N7—Ru2—N10	92.59 (12)	C14—C13—H13	120.1
N8—Ru2—N12	101.72 (13)	C15—C14—C13	118.0 (5)
N11—Ru2—N12	78.74 (14)	C15—C14—H14	121.0
N9—Ru2—N12	92.80 (13)	C13—C14—H14	121.0
N7—Ru2—N12	92.36 (13)	N3—C15—C14	123.5 (4)
N10—Ru2—N12	157.63 (13)	N3—C15—H15	118.3
F3—P1—F6	179.39 (15)	C14—C15—H15	118.3
F3—P1—F4	90.49 (14)	N4—C16—C17	122.5 (4)
F6—P1—F4	89.79 (14)	N4—C16—H16	118.8
F3—P1—F1	90.57 (13)	C17—C16—H16	118.8
F6—P1—F1	89.14 (13)	C18—C17—C16	119.5 (4)
F4—P1—F1	178.79 (15)	C18—C17—H17	120.2
F3—P1—F2	90.07 (13)	C16—C17—H17	120.2
F6—P1—F2	90.47 (13)	C17—C18—C19	118.8 (4)
F4—P1—F2	90.58 (14)	C17—C18—H18	120.6
F1—P1—F2	90.00 (13)	C19—C18—H18	120.6
F3—P1—F5	89.60 (13)	C20—C19—C18	120.1 (4)
F6—P1—F5	89.85 (13)	C20—C19—H19	120.0
F4—P1—F5	89.98 (14)	C18—C19—H19	120.0
F1—P1—F5	89.45 (13)	N4—C20—C19	120.7 (4)
F2—P1—F5	179.36 (16)	N4—C20—C21	113.8 (3)
F7—P2—F12	93.0 (5)	C19—C20—C21	125.0 (4)
F7—P2—F10'	129.4 (7)	N5—C21—C33	116.9 (3)
F7—P2—F9'	139.3 (7)	N5—C21—C20	112.4 (3)
F12—P2—F9'	127.3 (7)	C33—C21—C20	130.6 (4)
F10'—P2—F9'	90.0 (7)	N5—C22—C34	117.1 (4)
F10'—P2—F12'	90.3 (8)	N5—C22—C23	112.6 (3)
F9'—P2—F12'	168.2 (8)	C34—C22—C23	130.3 (4)
F7—P2—F9	90.7 (4)	N6—C23—C24	121.3 (4)
F12—P2—F9	171.6 (4)	N6—C23—C22	114.4 (3)
F10'—P2—F9	139.9 (7)	C24—C23—C22	124.0 (4)
F12'—P2—F9	129.7 (8)	C25—C24—C23	119.0 (4)
F7—P2—F11	87.6 (3)	C25—C24—H24	120.5
F12—P2—F11	87.2 (3)	C23—C24—H24	120.5
F10'—P2—F11	96.9 (5)	C26—C25—C24	119.7 (4)
F9'—P2—F11	98.7 (6)	C26—C25—H25	120.2
F12'—P2—F11	93.0 (4)	C24—C25—H25	120.2
F9—P2—F11	85.5 (2)	C25—C26—C27	118.8 (4)
F7—P2—F8	92.4 (3)	C25—C26—H26	120.6
F12—P2—F8	94.0 (3)	C27—C26—H26	120.6
F10'—P2—F8	84.1 (5)	N6—C27—C26	122.7 (4)
F9'—P2—F8	80.5 (6)	N6—C27—H27	118.7
F12'—P2—F8	87.8 (4)	C26—C27—H27	118.7
F9—P2—F8	93.3 (2)	N7—C28—C29	122.7 (4)
F11—P2—F8	178.79 (18)	N7—C28—H28	118.6
F12—P2—F7'	145.0 (7)	C29—C28—H28	118.6
F10'—P2—F7'	168.2 (6)	C30—C29—C28	119.2 (4)
F9'—P2—F7'	87.0 (7)	C30—C29—H29	120.4
F12'—P2—F7'	90.4 (8)	C28—C29—H29	120.4
F11—P2—F7'	94.9 (4)	C29—C30—C31	118.8 (4)
F8—P2—F7'	84.2 (4)	C29—C30—H30	120.6
F7—P2—F10	172.8 (4)	C31—C30—H30	120.6
F12—P2—F10	88.6 (5)	C32—C31—C30	120.2 (4)
F12'—P2—F10	143.2 (8)	C32—C31—H31	119.9
F9—P2—F10	86.9 (4)	C30—C31—H31	119.9
F11—P2—F10	85.6 (3)	C31—C32—N7	120.4 (4)
F8—P2—F10	94.4 (3)	C31—C32—C33	125.3 (4)
F7'—P2—F10	126.4 (7)	N7—C32—C33	113.8 (3)
F14—P3—F18	92.9 (2)	N8—C33—C21	117.1 (3)
F14—P3—F16	89.06 (19)	N8—C33—C32	112.9 (3)
F18—P3—F16	89.84 (19)	C21—C33—C32	130.0 (4)
F14—P3—F13	90.81 (18)	N8—C34—C22	117.3 (4)
F18—P3—F13	91.75 (18)	N8—C34—C35	112.8 (3)
F16—P3—F13	178.40 (19)	C22—C34—C35	129.8 (4)
F14—P3—F17	176.7 (2)	C36—C35—N9	120.9 (4)
F18—P3—F17	90.30 (19)	C36—C35—C34	125.1 (4)
F16—P3—F17	89.95 (17)	N9—C35—C34	113.8 (3)
F13—P3—F17	90.09 (16)	C35—C36—C37	120.1 (4)
F14—P3—F15	90.3 (2)	C35—C36—H36	120.0
F18—P3—F15	176.8 (2)	C37—C36—H36	120.0
F16—P3—F15	89.83 (17)	C36—C37—C38	119.3 (4)
F13—P3—F15	88.58 (16)	C36—C37—H37	120.4
F17—P3—F15	86.48 (18)	C38—C37—H37	120.4
F23—P4—F20	178.1 (8)	C39—C38—C37	118.5 (4)
F23—P4—F19	88.6 (7)	C39—C38—H38	120.8
F20—P4—F19	92.7 (6)	C37—C38—H38	120.8
F23—P4—F24	90.0 (7)	N9—C39—C38	122.9 (4)
F20—P4—F24	91.3 (7)	N9—C39—H39	118.5
F19—P4—F24	91.5 (7)	C38—C39—H39	118.5
F23—P4—F22	90.0 (6)	N10—C40—C41	122.3 (4)
F20—P4—F22	88.6 (6)	N10—C40—H40	118.9
F19—P4—F22	177.3 (8)	C41—C40—H40	118.9
F24—P4—F22	90.8 (7)	C42—C41—C40	118.9 (4)
F23—P4—F21	91.6 (8)	C42—C41—H41	120.6
F20—P4—F21	87.1 (6)	C40—C41—H41	120.6
F19—P4—F21	89.0 (8)	C41—C42—C43	119.8 (4)
F24—P4—F21	178.3 (9)	C41—C42—H42	120.1
F22—P4—F21	88.7 (8)	C43—C42—H42	120.1
F24'—P4'—F21'	177.5 (11)	C42—C43—C44	119.8 (4)
F24'—P4'—F23'	89.7 (9)	C42—C43—H43	120.1
F21'—P4'—F23'	88.2 (10)	C44—C43—H43	120.1
F24'—P4'—F19'	89.9 (9)	N10—C44—C43	120.2 (4)
F21'—P4'—F19'	88.7 (9)	N10—C44—C45	115.0 (4)
F23'—P4'—F19'	89.8 (8)	C43—C44—C45	124.8 (4)
F24'—P4'—F22'	88.7 (8)	N11—C45—C46	119.4 (4)
F21'—P4'—F22'	92.7 (9)	N11—C45—C44	113.3 (4)
F23'—P4'—F22'	91.0 (8)	C46—C45—C44	127.3 (4)
F19'—P4'—F22'	178.4 (11)	C47—C46—C45	119.2 (4)
F24'—P4'—F20'	90.9 (9)	C47—C46—H46	120.4
F21'—P4'—F20'	91.1 (9)	C45—C46—H46	120.4
F23'—P4'—F20'	179.1 (11)	C46—C47—C48	120.6 (4)
F19'—P4'—F20'	89.6 (8)	C46—C47—H47	119.7
F22'—P4'—F20'	89.7 (7)	C48—C47—H47	119.7
C1—N1—C5	117.9 (4)	C47—C48—C49	119.1 (4)
C1—N1—Ru1	128.1 (3)	C47—C48—H48	120.4
C5—N1—Ru1	113.9 (3)	C49—C48—H48	120.4
C10—N2—C6	121.5 (4)	N11—C49—C48	119.5 (4)
C10—N2—Ru1	119.0 (3)	N11—C49—C50	113.0 (3)
C6—N2—Ru1	119.2 (3)	C48—C49—C50	127.4 (4)
C15—N3—C11	118.7 (4)	N12—C50—C51	120.4 (4)
C15—N3—Ru1	127.5 (3)	N12—C50—C49	114.9 (4)
C11—N3—Ru1	113.8 (3)	C51—C50—C49	124.7 (4)
C16—N4—C20	118.4 (3)	C52—C51—C50	120.2 (4)
C16—N4—Ru1	126.4 (3)	C52—C51—H51	119.9
C20—N4—Ru1	114.7 (2)	C50—C51—H51	119.9
C22—N5—C21	123.1 (3)	C51—C52—C53	119.5 (5)
C22—N5—Ru1	118.0 (3)	C51—C52—H52	120.3
C21—N5—Ru1	118.9 (3)	C53—C52—H52	120.3
C27—N6—C23	118.3 (3)	C52—C53—C54	118.7 (5)
C27—N6—Ru1	127.7 (3)	C52—C53—H53	120.7
C23—N6—Ru1	113.5 (3)	C54—C53—H53	120.7
C28—N7—C32	118.2 (3)	N12—C54—C53	122.5 (4)
C28—N7—Ru2	127.0 (3)	N12—C54—H54	118.7
C32—N7—Ru2	114.6 (2)	C53—C54—H54	118.7
C34—N8—C33	123.2 (3)	N13—C55—C56	170.6 (17)
C34—N8—Ru2	118.4 (3)	N14—C57—C58	178.1 (6)
C33—N8—Ru2	118.4 (3)	N13'—C55'—C56'	172 (4)
C39—N9—C35	118.0 (3)	C55'—C56'—H56D	109.5
C39—N9—Ru2	127.3 (3)	C55'—C56'—H56E	109.5
C35—N9—Ru2	114.4 (3)	H56D—C56'—H56E	109.5
C40—N10—C44	119.0 (4)	C55'—C56'—H56F	109.5
C40—N10—Ru2	126.8 (3)	H56D—C56'—H56F	109.5
C44—N10—Ru2	114.2 (3)	H56E—C56'—H56F	109.5
C49—N11—C45	122.2 (4)	C57—C58—H58A	109.5
C49—N11—Ru2	119.1 (3)	C57—C58—H58B	109.5
C45—N11—Ru2	118.7 (3)	H58A—C58—H58B	109.5
C54—N12—C50	118.6 (4)	C57—C58—H58C	109.5
C54—N12—Ru2	127.2 (3)	H58A—C58—H58C	109.5
C50—N12—Ru2	114.1 (3)	H58B—C58—H58C	109.5
N1—C1—C2	122.9 (4)	N15—C59—C60	177.3 (7)
N1—C1—H1	118.6	C59—C60—H60A	109.5
C2—C1—H1	118.6	C59—C60—H60B	109.5
C3—C2—C1	119.0 (4)	H60A—C60—H60B	109.5
C3—C2—H2	120.5	C59—C60—H60C	109.5
C1—C2—H2	120.5	H60A—C60—H60C	109.5
C4—C3—C2	119.1 (4)	H60B—C60—H60C	109.5
C4—C3—H3	120.4	N16—C61—C62	178.7 (7)
C2—C3—H3	120.4	C61—C62—H62A	109.5
C3—C4—C5	120.1 (4)	C61—C62—H62B	109.5
C3—C4—H4	119.9	H62A—C62—H62B	109.5
C5—C4—H4	119.9	C61—C62—H62C	109.5
N1—C5—C4	120.9 (4)	H62A—C62—H62C	109.5
N1—C5—C6	115.0 (4)	H62B—C62—H62C	109.5
